# Characteristics and potentials of stem cells derived from human degenerated nucleus pulposus: potential for regeneration of the intervertebral disc

**DOI:** 10.1186/s12891-017-1567-4

**Published:** 2017-06-05

**Authors:** Xiao-Chuan Li, Yong Tang, Jian-Hong Wu, Pu-Shan Yang, De-Li Wang, Di-Ke Ruan

**Affiliations:** 1 Department of Orthopaedic Surgery, Navy General Hospital, Beijing, 100048 China; 2 Department of Orthopedic Surgery, Gaozhou people’s Hospital, No 89, Xi-Guan Road, Guangdong Guangzhou, 525200 China

**Keywords:** Intervertebral disc, Nucleus pulposus derived stem cells, Biological characteristics, Endogenous repair, Cell therapy

## Abstract

**Background:**

Eliminating the symptoms during treatment of intervertebral disc degeneration (IVDD) is only a temporary solution that does not cure the underlying cause. A biological method to treat this disorder may be possible by the newly discovered nucleus pulposus derived stem cells (NPDCs). However, the uncertain characteristics and potential of NPDCs calls for a comprehensive study.

**Methods:**

In the present study, nucleus pulposus samples were obtained from 5 patients with IVDD undergoing discectomy procedure and NPDCs were harvested using fluorescence activated cell sorting (FACS) by the co-expression of GD2^+^ and Tie2^+^. After in vitro expansion, the properties of NPDCs were compared with those of bone marrow mesenchyme stem cells (BMSCs) from the same subjects.

**Results:**

NPDCs performed similar properties in cell colony-forming ability, cell proliferation rate, cell cycle and stem cell gene expression similar to those of BMSCs. In addition, NPDCs could be differentiated into osteoblasts, adipocytes, and chondrocytes, and are found to be superior in chondrogenesis but inferior in adipocyte differentiation.

**Conclusions:**

NPDCs derived from the degenerated intervertebral disc still keep the regeneration ability similar to BMSCs. Besides, the superior capacity in chondrogenesis may provide a promising cell candidate for cell-based regenerative medicine and tissue engineering in IVDD.

## Background

A common health problem of the general population affecting both the individual’s quality of life and the family’s economic burden is lower back pain [1]. Intervertebral disc degeneration (IVDD) has been confirmed as the primary underlying reason strongly associated with lower back pain [2, 3]. Under normal circumstances, the nucleus pulposus (NP) can maintain its homeostasis and strike a balance between damage degeneration and regeneration repair [4]. Under pathological conditions, this homeostasis is disturbed by decreasing NP cell numbers and changing cell phenotype, which eventually leads to spinal instability, disc height loss, and bulging/prolapse [5]. To date, the main treatments for IVDD are focused on eliminating the symptoms rather than restoring normal function [6]. Besides this, these treatments may have several side effects like chronic pain, adjacent segment disease, and even revision surgery [7, 8]. Thus, it is necessary to highlight the critical urgency and importance of cellular therapy in this area.

The reduction of cell number leading to decreased extracellular matrix production may result in poor IVD resilience, decreased biomechanical properties, and eventual malfunction [1, 9]. Recent advances in the field of mesenchymal stromal cells (MSCs) offer promising applications in several other fields [10–12]. However, in IVDD, the harsh environment of high compressive load, acidic pH, hypoxia, hyperosmolarity, and limited nutrition makes it difficult for exogenous cells to survive and proliferate [13–15]. Nonetheless, hope arises in the discovery of nucleus pulposus derived stem cells (NPDCs), which may act as a candidate for endogenous repair in NP tissue [16, 17].

In the last few years, several groups have proposed the existence of stem cells in the NP tissue [18]. At First, Risbud et al [19] demonstrated the existence of NP progenitor cells in the NP and Henriksson et al [20] then found cell proliferation zones as a potential stem cell niche in the IVD region. Afterwards,, Blanco et al [18] reported that the NPDCs hold the same characteristic as BMSCs. Subsequently, many studies have transferred the studies in demonstrating the existence of NPDCs in other species [21–23]. However, whether the NPDCs from human degenerated NP tissues hold the regenerative potential urgently need to be comprehensively and systematically studied. In addition, all of previous studies reporting NPDCs were directly harvested from NP tissues which may also contain other types of cells [24]. Sakai et al [25] first reported that GD2 and Tie 2 could be used as identification markers for NPDCs. Therefore, in the present study, we obtained NPDCs by fluorescence-activated cell sorting (FACS) and compared them with BMSCs obtained from the same subject. Cell proliferation rate, colony formation ability, cell cycle, expression of stem cell genes, and the ability to differentiate into osteoblasts, adipocytes, and chondrocytes were compared between these two cell types. Through this study, we aimed to provide the molecular and cellular characterization of purified human NPDC subpopulations.

## Methods

### Case selection

The study was undertaken in the Department of Orthopedics of two hospitals (Navy General Hospital, Beijing, China, and Gaozhou people’s Hospital, Guangdong, China). The ethics committee of Navy General Hospital, Beijing, China and Gaozhou people’s hospital have approved this study and the procedures are in accordance with the Helsinki Declaration. Each patient enrolled in this study signed the informed consent. The NP tissue was obtained from 5 patients who underwent posterior discectomy surgery for lumbar degenerative disease. Meanwhile, bone marrow (BM) were also derived from the same patient respectively. The average age of the patients was 50.6 years (range: 45–56 years), and he details of all samples are shown in Table 1.

**Table 1:** Details characteristic of the enrolled patients

Case NO.	Diagnosis	Disc level	Modic type	BM(ml)	Gender	Age(year)
1	Spinal stenosis	L4-L5	II	7.0	M	54
2	Spinal stenosis	L5-S1	III	7.5	F	48
3	Spondylolisthesis	L4-L5	II	9.0	F	52
4	Lumbar disc herniation	L4-L5	I	8.5	M	45
5	Lumbar disc herniation	L5-S1	II	6.5	M	37

### Histomorphometry of human degenerated NP tissue

NP tissue were fixed in 10% formalin at room temperature for 24 h, embedded in paraffin and sectioned to 5-μm-thick sections. The tissue slices were stained with H&E to indicate their histological features as described previously [26].

### Isolation, sorting, and culture of NPDCs

NPDCs were isolated as previously described [25]. Briefly, human NP tissues were fragmented into pieces no larger than 1 mm^3^ and digested with 0.2% collagenase II (Sigma, USA)in DMEM/F12 for 6-8 h in a humidified atmosphere containing 5% CO_2_ at 37 °C. After centrifugation for 5 min at 1500 rpm, the pellet was cultured at a density of 10^6^ cells/cm^2^ in DMEM/F12 supplemented with 10% FCS and 10 units/ml penicillin-streptomycin in a humidified atmosphere containing 5% CO_2_ at 37 °C. FACS analysis and purification of cells was conducted as described previously [25]. The passage 2 cells were analyzed and sorted on a FACS Vantage cell sorter (BD Biosciences) using monoclonal antibodies purchased from eBioscience (BD, USA). The GD2 (BD Pharmingen; 14; G2a, 50 TESTs) and Tie2 (R&D Systems, clone 83715, 100 TESTs) monoclonal antibodies are used for analysis and purification. The isolated cells were cultured at a density of 10^5^ cells/cm^2^ in DMEM/F12 supplemented with 10% FCS and 10 units/ml penicillin-streptomycin in a humidified atmosphere containing 5% CO_2_ at 37 °C.

### Isolation and culture of BMSCs

MSCs from bone marrow (BM) were isolated from vertebral body of the same patients as previously described [27].. Briefly, BM aspirate (5–10 ml) was collected using a syringe containing 10,000 IU of heparin to prevent coagulation. BM mononuclear cells (MNCs) were isolated by density-gradient centrifugation (Percoll solution, Sigma, USA), resuspended, and cultured as BM-MNCs. All the BM-MNCs were cultured at a density of 10^6^ cells/cm^2^ in a humidified atmosphere containing 5% CO_2_ at 37 °C. After 48 h, non-adherent cells were removed and adherent cells were washed twice with PBS and cultured in culture medium containing DMEM/F12 supplemented with 10% FCS and 10 units/ml penicillin-streptomycin. After reaching 80% confluence, the cells were harvested with 0.25% trypsin/EDTA (HyClone, USA) for 2 min and subcultured at a ratio of 1:3.

### Measurement of cell proliferation capacity

To measure the proliferation capacity, the Cell Counting Kit-8 (CCK-8, Dojindo Laboratories, Japan) and the Passage 4 (P4) cells were used as described previously [27]. Briefly, 10 μL of CCK-8 solution was added to each well of a 96-well plate containing 5000 cells. After incubating the plate at 37 °C for 4 h, absorbance at 450 nm was measured using a microplate absorbance reader (BioRad, USA). Cell proliferation was tested on days 1, 3, 5, 7, 9, 11 and 13. A blank 96-well plate was used for the zero setting. All experiments were performed thrice.

### Colony-forming assay

To compare the colony-forming abilities of NPDCs and BMSCs, P4 populations were plated in cell culture dishes at various seeding densities (100 and 1000 cells/10 cm^2^). After culturing for 14 days, crystal violet staining was performed to count the colonies as previously described [28]. Briefly, cells were washed thrice with PBS, fixed in 4% paraformaldehyde for 15 min, stained with 0.1% crystal violet (Keygen Biotech, Beijing, China) for 15 min, and washed 3 times with PBS before counting the number of colonies. A colony containing less than 50 cells was ignored. Colony-forming efficiency was calculated by dividing the number of colonies by the initial number of adherent cells.

### Cell cycle assay

For cell cycle analysis, both NPDCs and BMSCs of P4 population were fixed in 75% chilled ethanol for 72 h at 4 °C. After washing twice with PBS, 1 ml propidium iodide (PI, Invitrogen, USA) staining solution and 50 μL RNase A stock solution (Invitrogen, USA) were added and incubated at 4 °C for 3 h. Measurements were performed on a FACSCalibur flow cytometer (BD, USA) using CellQuest software (BD, USA) and acquiring 10,000 cells per sample.

### Reverse transcriptase polymerase chain reaction (RT-PCR) and real-time quantitative reverse transcriptase polymerase chain reaction (qPCR) assay

Total RNA extraction was performed in both P4 cells as described previously [27]. The RT reaction (2 μL) was amplified in quadruplex by real-time PCR (ABI PRISM 7000) in a final volume of 25 μL, using the SYBR Green Master Mix reagent (Applied Biosystems, Foster City, CA). The specific primers used for stem cell genes, and osteogenic, adipogenic, and chondrogenic markers, are shown in Table 2. β-actin expression was used to normalize the expression of all the genes. For each cDNA sample, the cycle threshold (Ct) value of each target sequence was subtracted from the Ct value of the reference gene. RT-PCR was used to analyze the expression level of stem cell genes and qPCR was performed to compare the difference in the expression of osteogenic, adipogenic, and chondrogenic genes between groups of cells after induction for 4 weeks.

**Table 2:** The Primers for Real-Time Polymerase Chain Reaction

Target gene	Primers sequence
RUNX-2	sense 5’-ACGACAACCGCACCATGGT-3’
	antisense 5’-CTGTAATCTGACTCTGTCCT-3’
alkaline phosphatase (ALP)	sense 5’-TGGAGCTTCAGA AGCTCAACACCA-3’
	antisense 5’-ATCTCGTTGTCTGAGTACCAGTCC-3’
peroxisome proliferators-activated receptor 2 (PPAR-2)	sense 5’-CGAGGGCGATCTTGACAGGAA -3’
	antisense 5’-CAGGGGGGTGATGTGTTTGAAC- 3’
adipogenic protein (APP)	sense 5’-CTGTCCAAGTCCAACAGCAA-3’
	antisense 5’-ACGTTGGCAGCTTTACGTCT-3’
lipoprotein lipase (LPL)	sense 5’-TCCGCGTGATTGCAGAGAGAG-3’
	antisense 5’-TGCTGCTTCTTTTGGCTCTGACT-3’
aggrecan (Agg)	sense 5’-TGAGGAGGGCTGGAACAAGTACC-3’
	antisense 5’-GGAGGTGGTAATTGCAGGGAACA-3’
collagen II (Col II)	sense 5’-TTTCCCAGGTCAAGATGGTC-3’
	antisense 5’-TCACCTGGTTTTCCACCTTC-3’
SOX-9	sense 5’-TGGCCGAGATGATCCTAAAAATAA -3’
	antisense 5’-GCGCTTGGATAGGTCATGTTTGT-3’
SOX-2	sense 5’-CCCCTGTGGTTACCTCTTCCTC-3’
	antisense 5’-GGCCGCTCTGGTAGTGCTG-3’
NANOG	sense 5’-ACCCCGTTTCACTGTGTTAGC-3’
	antisense 5’-GACGGCAGCCAAGGTTATTAAA-3’
OCT4	sense 5’-GGCAAGCGATCAAGCAGCGAC-3’
	antisense 5’-GGGAAAGGGACCGAGGAGTAC-3’
β-actin	sense 5’-GTGGGGCGCCCCAGGCACCA-3’
	antisense 5’-CTTCCTTAATGTCACGCACGATTTC-3’

### Western blot assay of type II collagen and aggrecan

The samples were incubated with an extraction solution containing 1 ml 4 M guanidine chloride (GuCl), 0.1 M -6-aminohexanoic acid, 20 mM –benzamidine hydrochloride, 10 mM -EDTA, 5 mM -N-ethylmaleimide and 0.5 mM phenylmethanesulfonyl fluoride, pH 5.0 at 4 °C for 3 h. GuCl extracted dialysate was resuspended in 50 ml of sample buffer and analyzed by electrophoresis on a 6% (w/v) SDS-PAGE gels. Equal amounts of protein per lane (50 μg) were loaded and separated by electrophoresis. Protein was transferred to a polyvinylidene fluoride (PVDF) membrane by electroblotting. Rinse the membrane in water and block the blotted membrane in freshly prepared PBS containing nonfat dry milk (5%) for 60 min at room temperature with constant agitation. The membrane was incubated with the human type II collagen (1:5000, Sigma, USA) and aggrecan (1:5000, Sigma, USA) antibodies overnight at 4 °C with agitation. After washed the membrane three times with PBS, the secondary goat anti-mouse horseradish peroxidase (HRP)-conjugated anti-body was added and incubated overnight at 4 °C with agitation. Subsequently, the blots were washed 5 times with PBS containing 0.05% Tween 20 and the signal of bound antibodies was developed by enhanced chemiluminescence.

### Osteogenic induction and quantitation of mineral deposits

For osteogenic differentiation, 2.0 × 10^4^ P4 cells were cultured in human MSC osteogenic differentiation medium (Cyagen Biosciences, Guangzhou, China), changed every 3 days. Cells were fixed with 4% formaldehyde and stained with Alizarin red staining (Sigma, USA) for 15 min after 21 days of culture. The cells were then stained with alizarin red for 15 min at 37 °C and washed thrice with distilled water. Finally, alizarin red staining was observed under a microscope (Leica, Germany).

### Adipogenic induction and quantitation of adipogenic capacity

Adipogenic differentiation was performed in cultures using induction medium A and maintenance medium B (Cyagen Biosciences, Guangzhou, China). The detailed contents of medium A and B have been described previously [22]. First, the 2.0 × 10^4^ P4 cells in each plate were incubated in induction medium A for 72 h, which was then replaced with maintenance medium B for 24 h. This 96 h induction-maintenance cycle was repeated four times and in the end, cells were incubated in maintenance medium B for 7 days. Cells cultured with culture medium only served as controls. To visualize the lipid-rich vacuoles in cells, oil red O staining was performed as described previously [27]. In short, cells were fixed with 4% formaldehyde and stained with oil red O for 15 min. Hematoxylin was used for nuclear staining. Finally, the cultures were extensively washed with distilled water to remove the excess stain.

### Chondrogenic induction and quantitation of chondrogenic capacity

Chondrogenic differentiation was performed as described previously [22]. Briefly, 2.0 × 10^4^ P4 cells were harvested and seeded into multi-well cell culture plates in culture medium. After 24 h, the culture medium was replaced with chondrogenic differentiation medium (Cyagen Biosciences, Guangzhou, China). Cells were treated twice weekly with fresh chondrogenic differentiation medium for 4 weeks. Cells cultured with culture medium only served as controls. The alcian blue intensity assay was performed as described previously [27]. Cells were treated twice weekly with fresh chondrogenic differentiation medium for 4 weeks. At last, cells were rinsed thrice with PBS, fixed with 4% paraformaldehyde for 15 min at room temperature, then washed with PBS, and stained with 1% alcian blue for 15 min.

### Statistical analysis

Colony formation, cell proliferation, percentage of cell cycle phases, stem cell gene expression levels, and the ability to differentiate into osteoblasts, adipocytes, and chondrocytes was reported as mean ± SD (standard deviation) values. The histological and western blot data are described qualitatively and shown as images. To quantify multiple differentiation capacities by morphology, the differentiation value (DV) method was adopted [29]. Briefly, five random high power fields from one source were counted using Image-Pro Plus version 6.0 (IPP 6.0). Quantifiable data comparing NPDCs and BMSCs were analyzed using Student’s *t* tests. All data analyses were performed using SPSS version 15.0. *p* < 0.05 was regarded as statistically significant.

## Results

### Gross morphology and histomorphology of human degenerated NP tissue

**Fig. 1:**
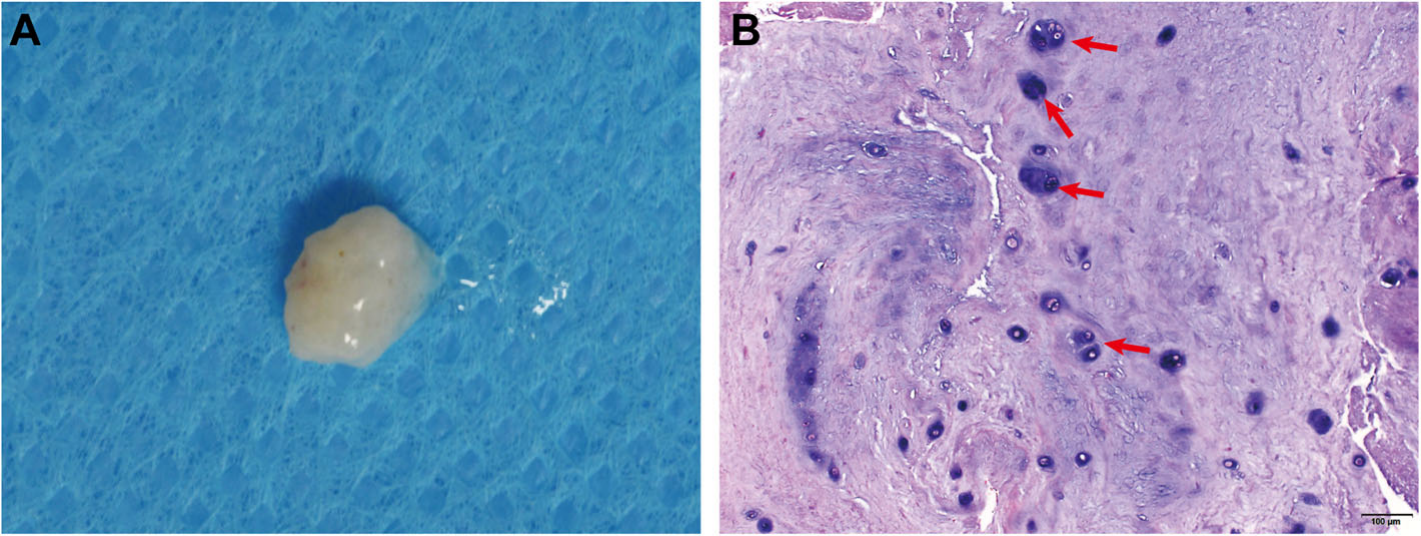
Gross morphology and histomorphology of degenerated NP tissue. **a**: gross morphology; **b**: histologic morphology with HE staining (Bar = 100 μm)

NP was white and translucent after being cleaned and no vascular or fat tissues were observed in any of the NP tissues (Fig. 1a). The extracellular matrix (ECM) of NP tissues was homogeneous and contained round-shaped cells distributed in different layers. (Fig. 1b). Besides, a proportion of highly proliferation cells was observed in NP tissue as shown in red arrows (Fig. 1b).

### FACS results

**Fig. 2:**
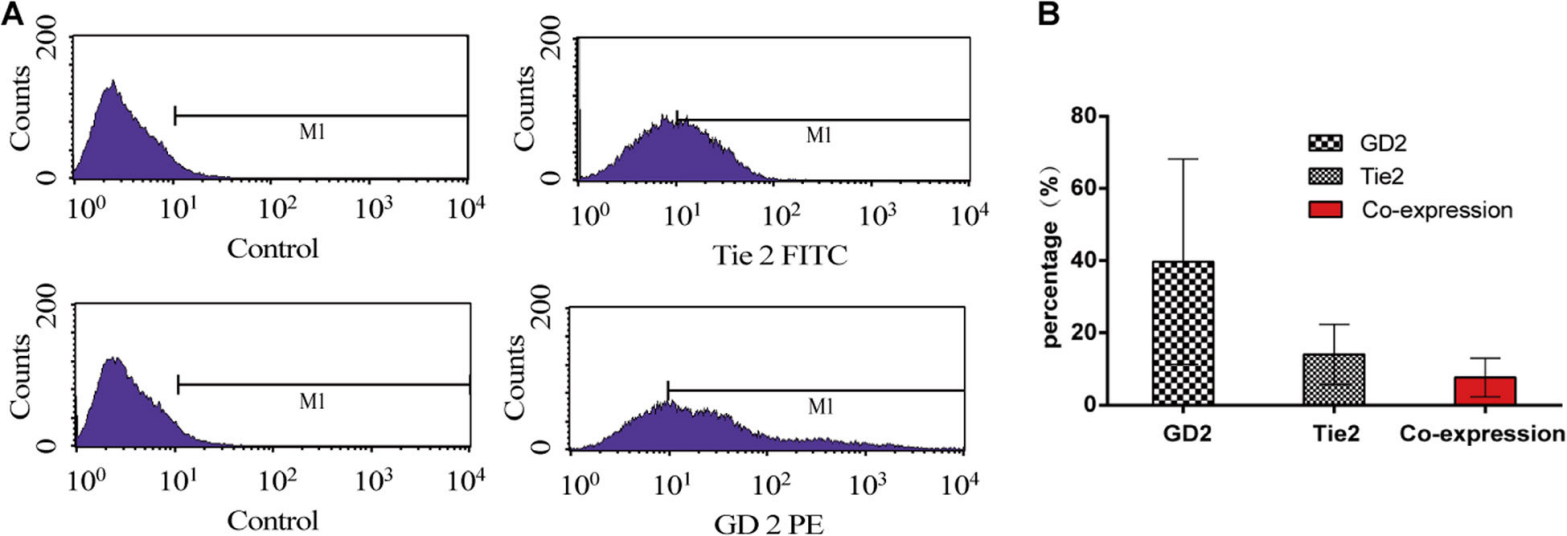
The positive expression of GD2 or Tie2 cells in degenerated NP derived cells. **a**: representative histogram of flow cytometry; **b**: cell percentage of positive expressing cells

For the NP-derived cells from each of the 5 patients, evaluation of GD2 and Tie2 positivity was performed by FACS. The results showed the presence of a proportion of cells, which expressed GD2 and Tie2 cells (39.8 ± 27.4%, 14.07 ± 8.3% and co-expression of 7.7 ± 5.4% Fig. 2). The cells were then sorted by FACS with double positive markers and the harvested cell population were seeded at a density of 10^5^ cells/cm^2^.

### Colony-forming abilities and proliferation capacity

**Fig. 3:**
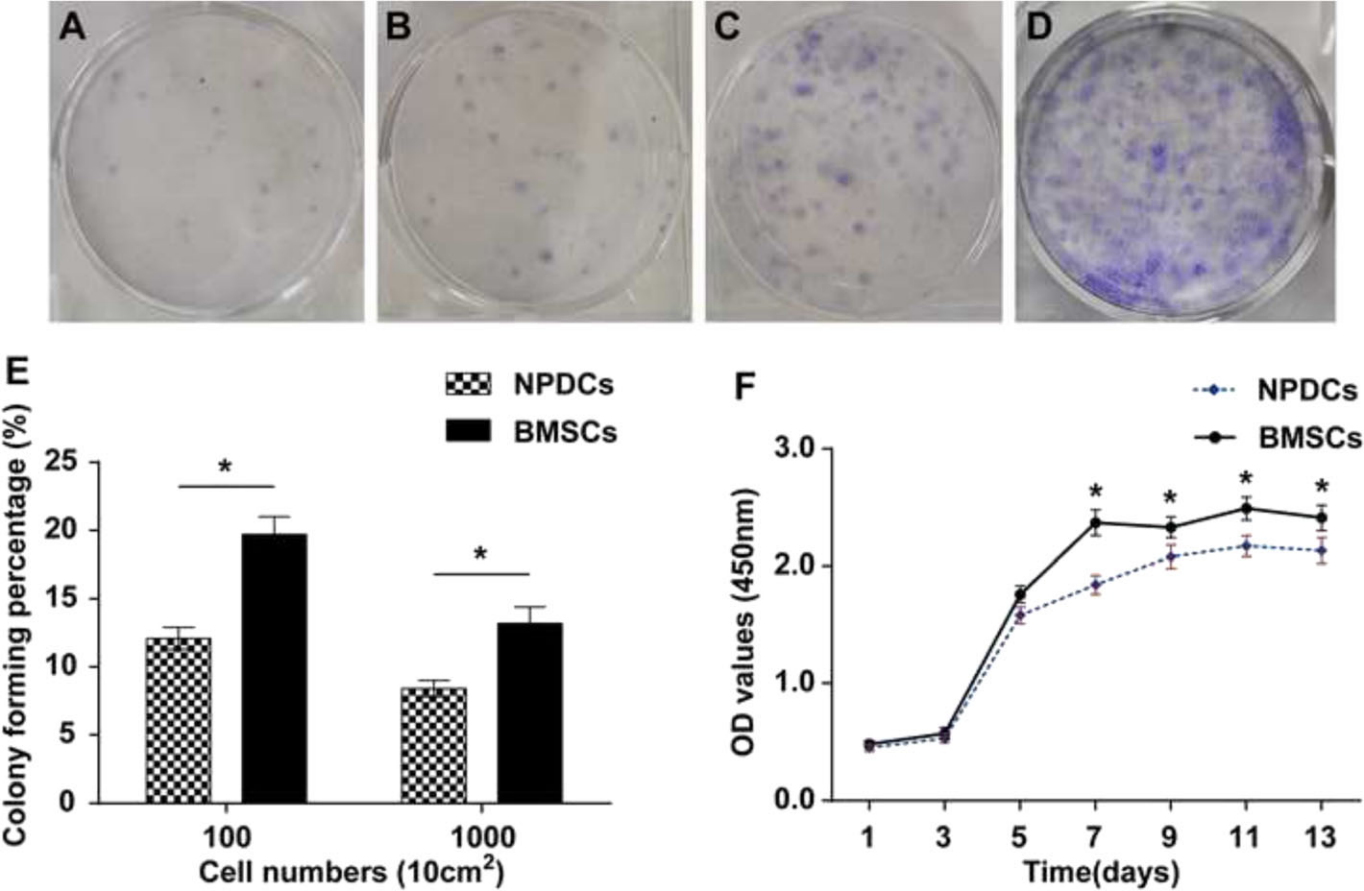
Colony formation and proliferation of NPDCs and BMSCs. The colonies stained with crystal violet at 13 days (**a**, **c**: NPDCs; **b**, **d**: BMSCs). Colony formation assay for NPDCs and BMSCs (**e**). The proliferation capacity of BMSCs and NPDCs measured by the CCK-8 assay (**f**). * *p* < 0.05. Data represents cells derived from 5 different patients (mean ± SD)

Two different cell densities were seeded to test the colony-forming abilities of both cells revealing that NPDCs and BMSCs could form colonies after 2 weeks (Fig. 3a-d). With seeding densities of 100 cells/10 cm^2^ and 1000 cells/10 cm^2^, BMSCs performed better colony formation (*p* < 0.05, Fig. 3e). Regarding proliferation capacity, both two groups exhibited similar growth tendencies. When the OD values were measured, a continuous increase was observed from day 1 to day 13 and a plateau period was formed from day 7–13. However, a slightly higher proliferation capacity was found in BMSCs at the last 4 time points (*p* < 0.05, Fig. 3f).

### Stem cell gene analysis

**Fig. 4:**
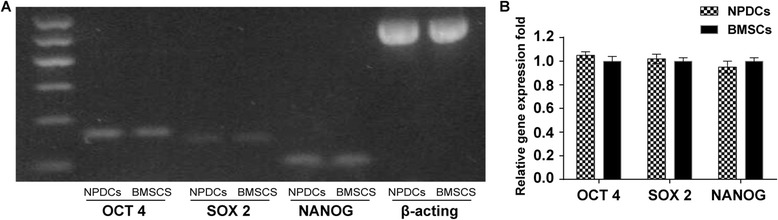
Stem cell genes (OCT-4, NANOG, and SOX-2) were expressed in both NPDCs and BMSCs. **a**: RT-PCR; **b**: qPCR

*OCT-4*, *NANOG*, and *SOX-2* are genes that are commonly expressed in stem cells. Both NPDCs and BMSCs were used to determine the expression of these genes and the results were similar in PT-PCR evaluation (Fig. 4a). In qPCR analysis, NPDCs showed gene expression levels that were comparable with those of BMSCs (*p* >0.05, Fig. 4b).

### Cell cycle assay

**Fig. 5:**
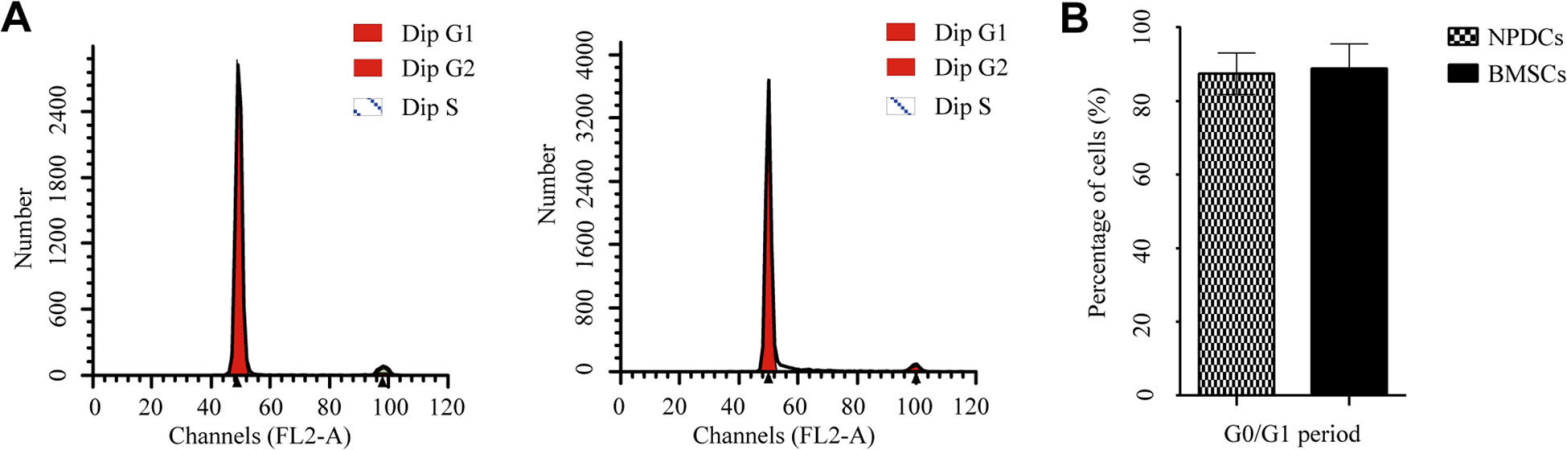
Cell cycle analysis of NPDCs and BMSCs. The cell cycle histograms of NPDCs and BMSCs are shown (**a**). There were no significant differences in G0/G1 cell cycle phases between the NPDCs and BMSCs (**b**). Data represents cells derived from 5 different patients (mean ± SD)

The percentage of cells in each phase of the cell cycle was analyzed by flow cytometry. Cell cycle analysis was conducted by measuring the DNA content from both stem cells. Approximately 90% of the NPDCs and BMSCs were in the G0/G1 phase (88.62% vs. 91.35%), and no significant differences were detected between both groups in this criterion(*p* > 0.05, Fig. 5).

### Osteogenic induction and related quantitative assays

**Fig. 6:**
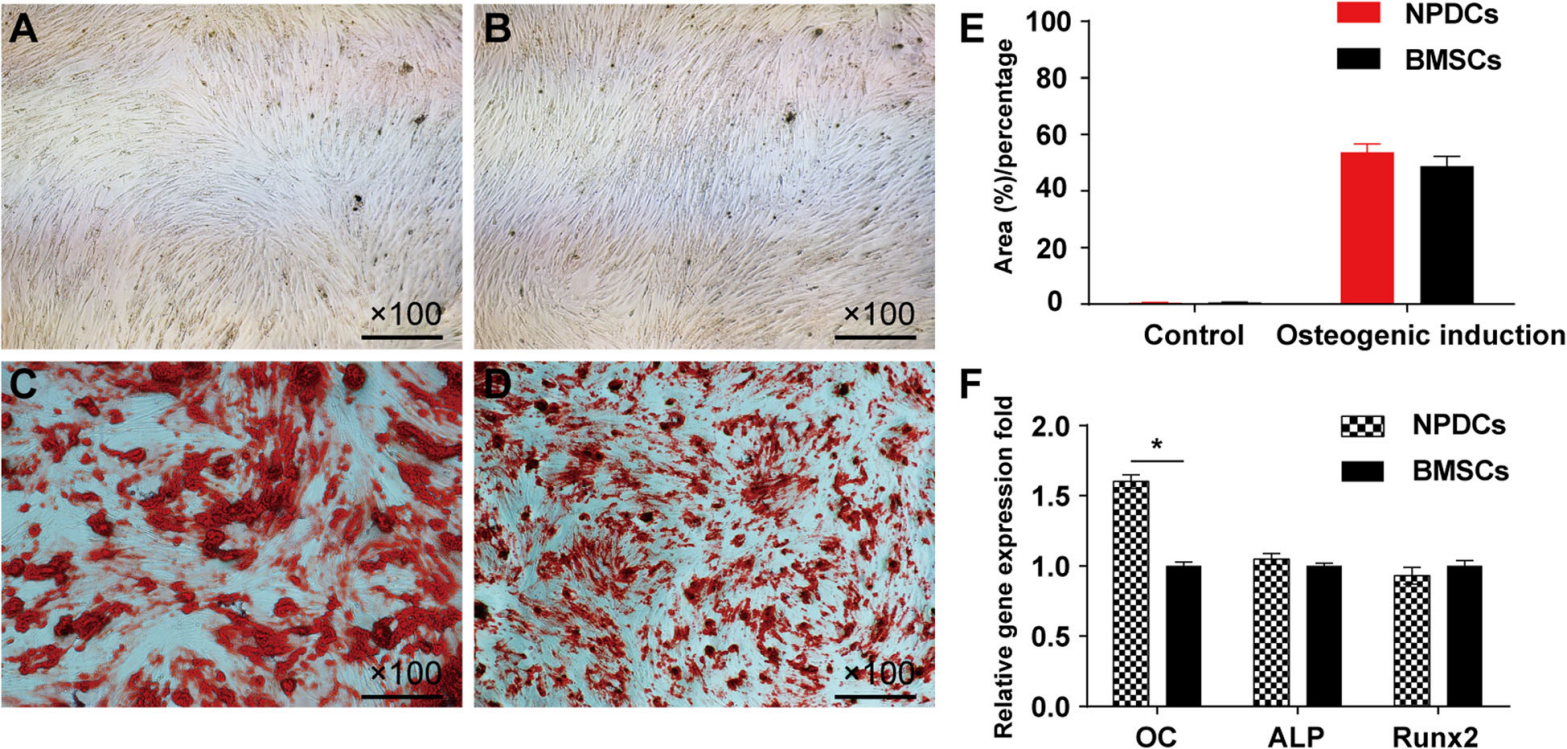
Osteogenic differentiation of NPDCs and BMSCs stained with *alizarin red* after 4 weeks. **a**: NPDCs; **b**: BMSCs; **c**: NPDCs after 4 weeks osteogenic induction; **d**: BMSCs after 4 weeks osteogenic induction. Quantitative analysis of mineral deposition in both cell types cultured in osteogenic medium showed no difference after 4 weeks (**e**). Higher expression levels were observed for OC mRNA in NPDCs, whereas no significant difference was observed in ALP and RUNX2 expression after 4-week induction (**f**). * *p* < 0.05. Data represents cells derived from 5 different patients (mean ± SD)

Osteogenic differentiation was confirmed by the deposition of an alizarin red positive mineralized matrix. From the morphology observed, the area percentage showed of no difference between the two groups after 4 weeks induction (Fig. 6a-d). The positively stained mineralized nodules in the NPDC group were larger and the qPCR analysis showed no significant difference in the expression of RUNX-2 and ALP genes between groups after 4 weeks(*p* >0.05, Fig. 6e, f). However, expression of the OC gene in the NPDCs was slightly higher (*p* < 0.05, Fig. 6f).

### Adipogenic differentiation and related quantitative assays

**Fig. 7:**
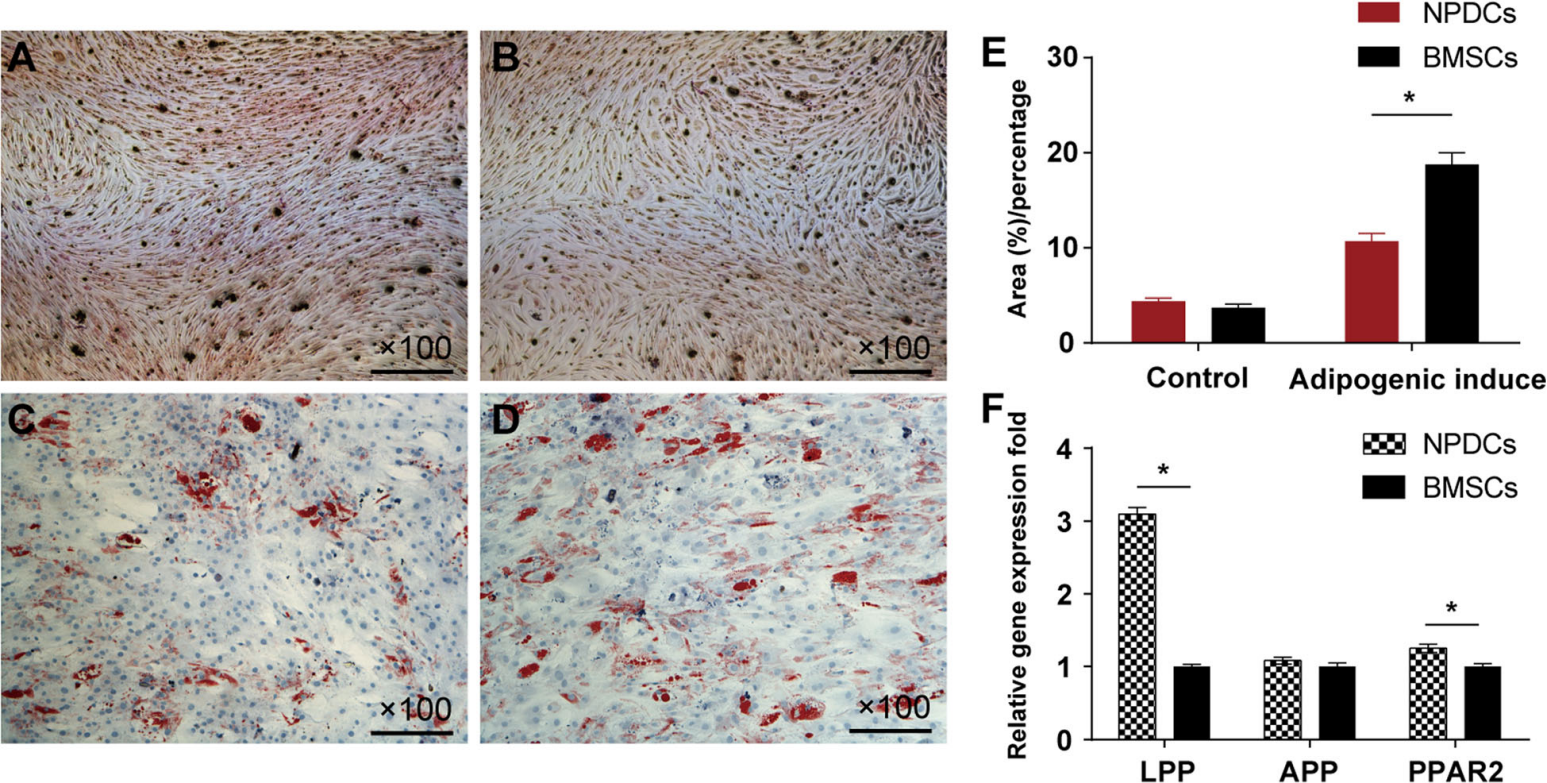
Adipogenic differentiation of NPDCs and BMSCs stained with *oil red O* after 4 weeks. **a**: NPDCs; **b**: BMSCs; **c**: NPDCs after 4 weeks adipogenic induction; **d**: BMSCs after 4 weeks adipogenic induction. Quantitative analysis of lipid-rich vacuoles in both two cell types showed superior adipogenic potential in BMSCs **e** The mRNA levels of adipogenic genes showed lower expression levels of LPP and PPAR2 in NPDCs after 4-week induction compared with BMSCs (**f**). * *p* < 0.05. Data represents cells derived from 5 different patients (mean ± SD)

The observation of lipid-rich vacuoles stained with oil red O was used to evaluate adipogenic ability. Both cell types exhibited intracellular lipid vacuoles after 4 weeks of induction. The area that was positive for oil red O accumulated and superior adipogenic differentiation capability was detected in BMSCs after 4 weeks (*p* < 0.05, Fig. 7a-e). In addition, qPCR analysis of adipogenic mRNA (*PPAR-2*, *LPP*, and *APP*) showed higher expression of *PPAR-2* and *LPP* in BMSCs (Fig. 7f).

### Chondrogenic differentiation and related quantitative assays

**Fig. 8:**
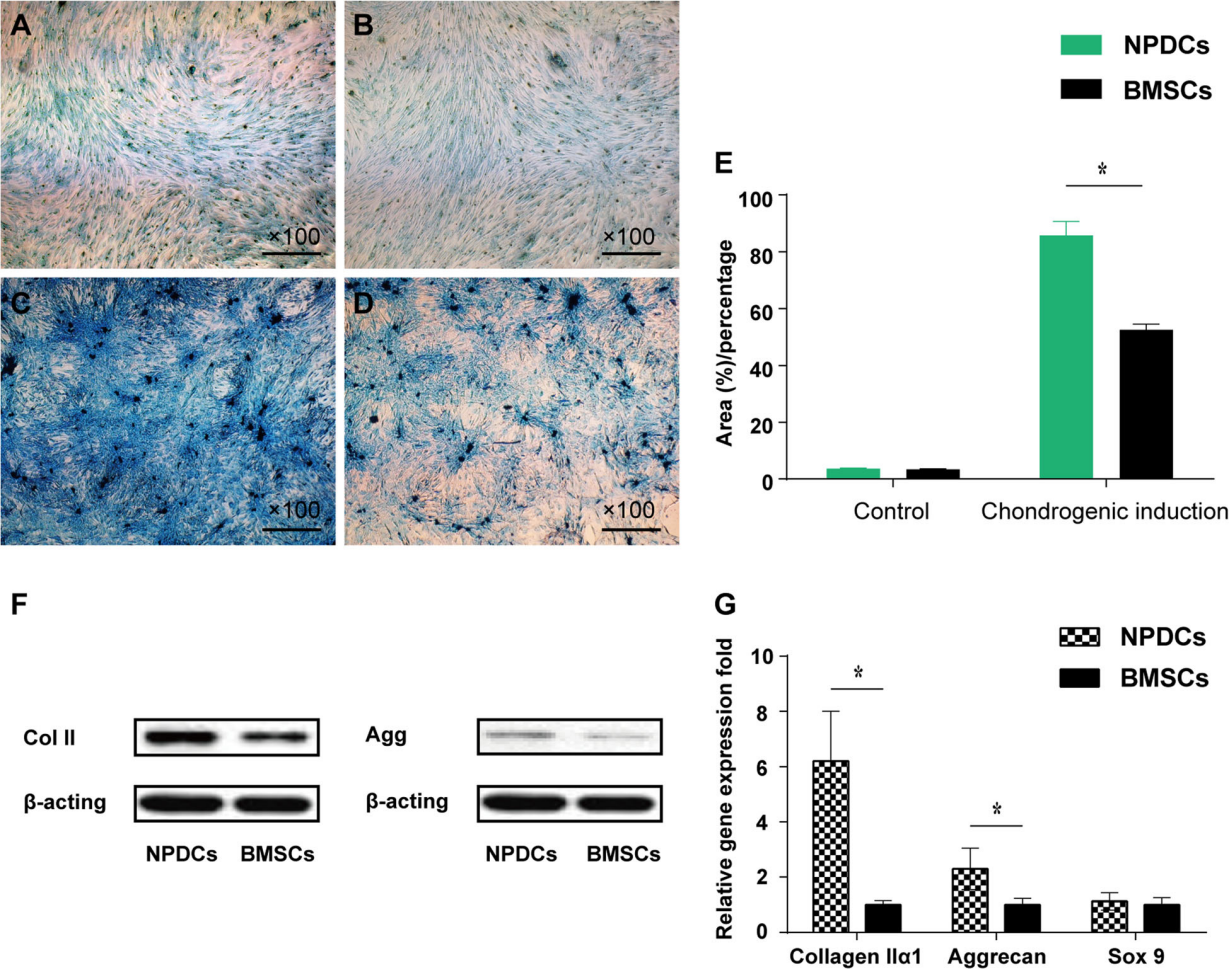
Chondrogenic differentiation of NPDCs and BMSCs stained with *Alcian blue* after 4 weeks. **a**: NPDCs; **b**: BMSCs; **c**: NPDCs after 4 weeks chondrogenic induction; **d**: BMSCs after 4 weeks chondrogenic induction. Larger positive *alcian blue* area were detected in NPDCs after 4 weeks chondrogenic induction (**e**) and Higher mRNA expression level of Collagen IIα1and Aggrecan were observed in NPDCs after 4-week induction **g** Higher Col II and aggrecan protein levels were found by western blotting in NPDCs (**f**). *p < 0.05. Data represents cells derived from 5 different patients (mean ± SD)

For in vitro chondrogenesis, the Alcian blue intensity assay was performed to evaluate chondrogenic potential. The area that was positive for Alcian blue from NPDCs were larger than BMSCs after 4 weeks of induction (*p* < 0.05, Fig. 8a-e). Regarding the expression of chondrogenic genes, higher mRNA expression levels of collagen IIα1 and aggrecan were observed in NPDCs after 4 weeks of induction (Fig. 8g). Western blot analysis indicated that both Collagen II (Col II) and aggrecan (agg) protein levels were higher in NPDCs, which was in accordance with qPCR results (Fig. 8f).

## Discussion

This report describes the isolation of human NPDCs by FACS and their comprehensive in vitro characterization compared to those of BMSCs. Thus, the results of this study may play a helpful role in intervertebral disc tissue engineering and regeneration. In this study, the morphology, proliferation potential, colony formation ability, cell cycle, stem cell gene expression, and potential for multiple lineage differentiation were assessed for NPDCs and BMSCs from the same subjects. Our study reveals that the sorted NPDCs possess the same characteristics as those of BMSCs in most respects but show superior ability for chondrogenic differentiation in vitro. These findings provide comprehensive evidence of a new cell resource with more prospects in IVDD tissue engineering.

Our study is based on previous other studies describing the presence of stem cells derived from NP tissue [18, 19, 22, 23, 30–32]. However, studies on the cell characteristics and potential of these cells are lacking. Although NPDCs are proposed as a valid and promising cell source in disc regenerated tissue engineering, sufficient evidence for this is crucially needed. Additionally, all the published studies were focused on the existence of NPDCs by directly harvesting them from the NP tissue [18, 20, 23, 30, 32, 33], which is possibly a mixture of impure and polymictic cells. In this study, we isolated NPDCs through FACS and expanded these cells in vitro [26]. Therefore, our stem cells were purified and credible.

Currently, MSCs are confirmed according to the guidelines of the International Society for Cellular Therapy (ISCT) [34], which include in vitro adherence to plastic surfaces, immunophenotypic profile, and multilineage differentiation capacity into osteogenic, adipogenic, and chondrogenic lineages. However, with respect to immunophenotypic profile, it is not known whether the stem cells derived from NP would conform to these requirements. Therefore, due to lack of specific immunophenotypic profile of NPDCs, all previously reported NPDCs were considered by ISCT as the reference standard. In this study, NPDCs were sorted by FACS, and so the immunophenotypic profile of the cells included the expression of GD2 and Tie2, which was reported to be more suitable for NPDCs [25]. Moreover, because ISCT criteria for defining MSCs is minimal and limited, several other potential abilities were additionally tested for a comprehensive evaluation in this study.

From gross morphology, NP retrieved samples showed white gelatin tissue without other potential sources like vascular or fat cells ensuring that other potential cells were excluded. Meanwhile, from the histological aspect, a proportion of highly proliferating cells was observed in NP tissue as shown with red arrows. This is in accordance with the reports demonstrating that the cell clusters attempting to restore matrix synthesis and mechanical function performed according to the characteristics of stem cells [20, 22, 35].

NPDCs adhered to the plastic surface of a tissue culture flask and were morphologically similar to BMSCs. However, the BMSCs showed slightly superior performance with respect to proliferation capacity and colony-forming abilities. Because the NPDCs used in this study were all harvested from the degenerated NP, it is possible that cell characteristics and potential was affected to some extent. Considering these data, we may conclude that NPDCs presented with similar stem cell properties as those of BMSCs, thus providing evidence for the presence of stem cells in degenerated NP and revealing the potential ability of this new source of stem cells.

In addition, NPDCs and BMSCs shared the same cell cycle characteristics and stem cell gene expression. It is reported that the vast majority of primitive progenitor cells reside in the G0/G1 phase [36]. In our study, the cell cycle assays showed that the major percentage of cells in both cell types were in the G0/G1 phase and less than 10% of the cells were in the S and G2 + M phases. Stem cell genes like OCT-4, NANOG, and SOX-2 are reported to be highly expressed in pluripotent cells and are considered as markers of primitive stem cells [37]. Thus, the equal expression of all three genes in both cell types indicated their similarity in this aspect.

Although NPDCs shared many characteristics with BMSCs, it is believed that adult MSCs originating from a certain tissue preferentially differentiate into the type of cells residing in this tissue [38, 39]. Our results are also in accordance with these findings. In osteogenic differentiation, although qPCR results showed that NPDCs had higher expression of OC mRNA, which is considered as a marker of mature osteoblasts [40], the positively stained area at different induction times showed no difference between both groups. Therefore, this result suggests that both cells have the same osteogenic induction potential.

Regarding adipogenic capacity, BMSCs exhibited a better adipogenic capacity than NPDCs both in DV assay and in qPCR analysis. LPP and PPAR2 expression levels were significantly higher in BMSCs after 3 and 4 weeks of induction. These findings were also supported by the previous study [18].

Finally, we quantitatively evaluated the chondrogenic potential of NPDCs and BMSCs. qPCR analysis showed higher expression of matrix transcripts (collagen II and aggrecan) in NPDCs. Furthermore, western blot indicated that protein expression of both Col II and aggrecan was higher in NPDCs, which was in accordance with the qPCR results. In conclusion, all the results demonstrated that NPDCs had a better chondrogenic potential than BMSCs.

The degenerated NP might contain a substantial amount of inflammation factors and cytokines that could have probably affected the cell properties in this study [41]. Moreover, the microenvironmental factors in the degenerated NP tissue and culture medium may affect the cell state and phenotype in some extend. Additionally, the NP samples used in our study were degenerated, and must have some difference in comparison with normal samples. Furthermore, considering this study was performed in vitro, the interactions between stem cells and their surrounding niche in vivo may affect the result of this study.

## Conclusions

In conclusion, this study reports the isolation of NPDCs from degenerated human NP and their comparison with BMSCs. NPDCs derived from the degenerated intervertebral disc still keep the regeneration ability similar to BMSCs. Based on this, an investigation into the characteristics and potential of NPDCs may provide a promising cell candidate for IDD regenerative medicine.

## Abbreviations

Agg: Aggrecan
ALP: Alkaline phosphatase
APP: Adipogenic protein
BM: Bone marrow
BM-MNCs: BM mononuclear cells
BMSCs: Bone marrow mesenchymal stem cells
CCK-8: Cell counting Kit-8
Col II: Collagen II
Ct: Cycle threshold
DMEM: Dulbecco’s modified eagle’smedium
DV: Differentiation value
ECM: Extracellular matrix
ECM: Extracellular matrix
FACS: Fluorescence-activated cell sorting
FCS: Fetal calf serum
FITC: Fluorescein isothiocyanate
IDD: Intervertebral disc degeneration
ISCT: International society for cellular therapy
LPL: Lipoprotein lipase
MSCs: Mesenchymal stromal cells
NP: Nucleus pulposus
NPDCs: Nucleus pulposus derived stem cells
OC: Osteocalcin
OD: optical density
PBS: Phosphate-buffered saline
PE: Phycoerythrin
PPAR-2: Peroxisome proliferators-activated receptor 2
SOX-9: SRY-box 9
